# Structural Determinants of PARP1 Selectivity from Molecular Dynamics Analysis of PARP1 and PARP2 Complexes

**DOI:** 10.3390/molecules31101592

**Published:** 2026-05-09

**Authors:** Dmitrii O. Shkil, Natalia A. Chesnokova, Andrey A. Ivashchenko, Elena V. Petersen, Philipp Y. Maximov

**Affiliations:** Institute of Future Biophysics, Institutsky Lane, 9, Dolgoprudny 141700, Moscow Region, Russia; na.chesnokowa@gmail.com (N.A.C.); ai@chemrar.ru (A.A.I.); petersen.ev@mipt.ru (E.V.P.); philipp.maximov@gmail.com (P.Y.M.)

**Keywords:** PARP1, PARP2, PARP1 selective inhibitors, molecular dynamics, protein–ligand interactions, saruparib, palacaparib

## Abstract

Selective inhibition of poly(ADP-ribose) polymerase 1 (PARP1) may reduce the hematologic toxicity associated with dual PARP1/PARP2 inhibition. We performed molecular dynamics simulations for five selective inhibitors in complexes with PARP1 and PARP2, using three independent 50 ns runs per complex after docking and equilibration, followed by protein–ligand interaction fingerprint and statistical analyses. All complexes remained dynamically stable, with ligand root-mean-square deviation values generally within 0.3 nm. Comparative analysis identified three αF-helix residue pairs with nominally reduced interaction frequencies in PARP2: Asn767/Ala336, Leu769/Gly338, and Asp770/Asp339 (*p* < 0.05). After Benjamini–Hochberg correction for multiple comparisons, Leu769/Gly338 remained significant (q < 0.05), indicating that this pair represents the most statistically robust interaction difference within this region. Using palacaparib as the most selective inhibitor, these differences were associated with weakened or lost hydrophobic, van der Waals, and cation–π interactions in PARP2. Selective binding of modern PARP1 inhibitors appears to be associated with αF-helix-dependent interaction patterns, providing a mechanistic basis for the rational design of next-generation selective inhibitors with improved selectivity and potentially reduced toxicity.

## 1. Introduction

The continuous process of DNA replication is accompanied by a large amount of damage, which includes nucleotide damage or nucleotide pair damage, cross-link formation, and DNA strand breaks (single-stranded (SSB) and double-stranded (DSB)). One of the important roles of DNA damage repair is played by the poly(ADP-ribose)polymerase (PARP) family of proteins [1,2].

**Figure 1:**
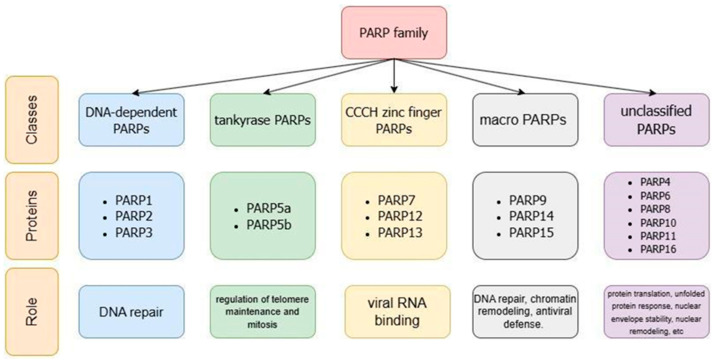
Classification and properties of PARP protein family.

Humans express 17 PARPs, identified on the basis of sequence homology to the catalytic domain of PARP1 [3,4]. The PARP family is further grouped into four subfamilies based on the presence of functionally characterized domains in regions outside the PARP domain (Figure 1): DNA-dependent PARPs, originally thought to require DNA binding for enzymatic activity (including PARP1, PARP2, PARP3); tankyrase PARPs, with ankyrin repeat protein-binding proteins (PARP5a, PARP5b); CCCH zinc finger PARPs, which contain CCCH zinc finger domains that have been shown to bind viral RNA (PARP7, PARP12, PARP13); and macro PARPs with ADPr-binding macrodomains (PARP9, PARP14, PARP15). The remaining PARPs are referred to as unclassified PARPs (including PARP4, PARP6, PARP8, PARP10, PARP11, PARP16) [3].

**Figure 2:**
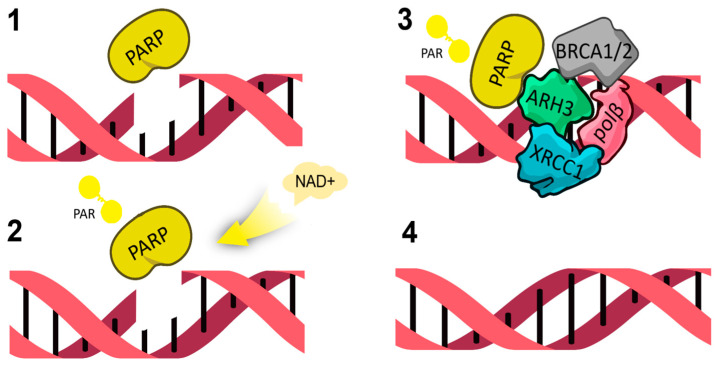
Mechanism of PARP DNA reparation. **1**—PARP1 binds to regions of DNA damage PAR. **2**—PARP1 uses NAD+ to create poly-(ADP-ribose) (PAR) polymers. **3**—The proteins move to the site of DNA damage, initiating the repair complex. **4**—DNA repair [6].

DNA-dependent PARPs have a similar mechanism for DNA damage repair. Acting as a “molecular sensor”, PARP1 is activated upon detection of single-strand breaks and binds to regions of damage via its DNA-binding “zinc finger” domain. After binding to damaged DNA, PARP1 increases its catalytic activity, uses NAD+ to create poly-(ADP-ribose) (PAR) polymers and transfers them to acceptor proteins [3]. This poly-ADP-ribosylation is a signal for various other proteins to move to the site of DNA damage, initiating the repair complex. After ADP-ribosylation, PARP1 undergoes molecular changes that eventually lead to a decrease in its affinity for DNA. It is released, ‘opening’ chromatin and giving access to the damaged area to proteins of the repair complex, which includes proteins such as ARH3 (Lig3 (DNA ligase 3), polβ, XRCC1, BRCA1/2 and others [5]. Upon initiation of the repair complex, PAR binds XRCC1 protein. PAR regulates the binding of histone H1 to chromatin, thereby attenuating chromatin compaction. The enzyme poly (ADP-ribose)-glycohydrolase (PARG) then removes the poly (ADP-ribose)-polymer from PARP, thereby reactivating it (Figure 2).

**Figure 3:**
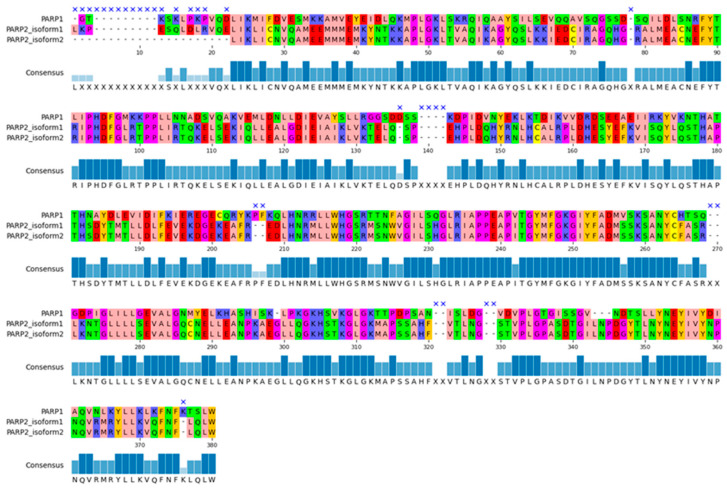
Alignment of the primary sequence of the catalytic domains of PARP1 and two isoforms of PARP2. The cross marks the gaps in sequences, the consensus shows the most frequent amino acid for a given position.

**Figure 4:**
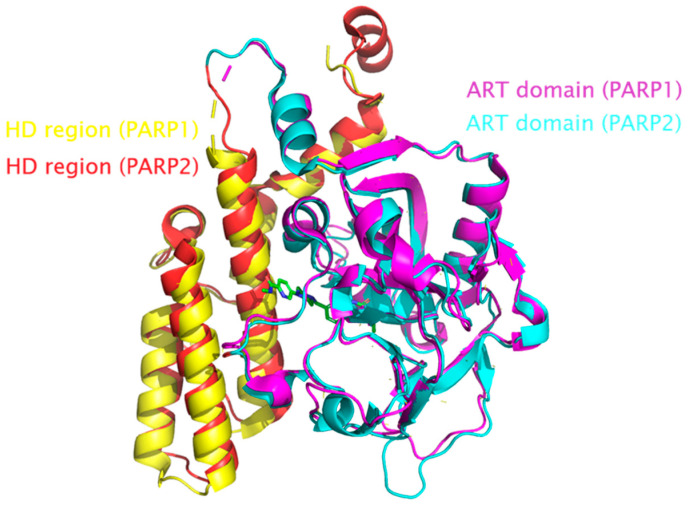
Superimposition of the 3-D structure of the catalytic domain of PARP1 (PDB: 7ONT) and PARP2 (PDB: 4TVJ). The HD region of PARP1 is shown by yellow, the HD region of PARP2—by red. The ART domain of PARP1 is shown by purple, the ART domain of PARP2—by blue. Saruparib is shown in green.

The best known and most widely expressed members of this group are the PARP1 and PARP2 proteins. They share similar sequences and a high degree of homology within their catalytic domains. As shown in Figure 3, the sequence similarity between PARP1 and the two PARP2 isoforms is approximately 50%. They also share a similar structural organization consisting of the autoinhibitory helical subdomain (HD) and the ADP-ribosyltransferase (ART) subdomain (Figure 4). The ART subdomain interacts with NAD+ and catalyzes ADP-ribosylation, whereas the HD inhibits PARP1 binding to NAD+ when PARP1 is in the non-DNA-bound state [7].

**Figure 5:**
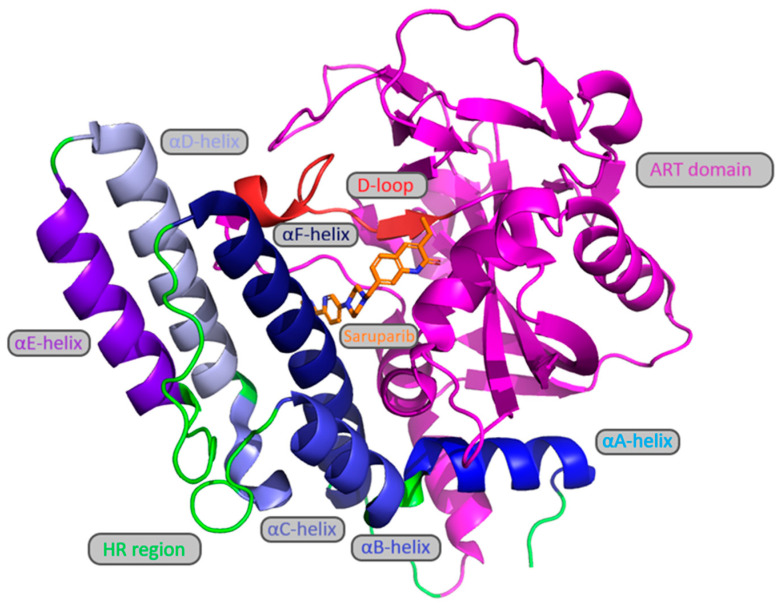
Structure of the catalytic domain of PARP1 (PDB: 7ONT).

The structure of the catalytic domain requires more detailed examination. The HD region consists of six α-helices, designated by letters of the Latin alphabet, which together form a hydrophobic core (Figure 5). The αF-helix is located adjacent to the NAD+ donor binding site. The ART domain contains both acceptor and donor sites. One of its important structural elements is the D-loop (Figure 5), which helps align the donor site and, in part, the acceptor site. The D-loop is highly conserved between PARP1 and PARP2 [8].

**Figure 6:**
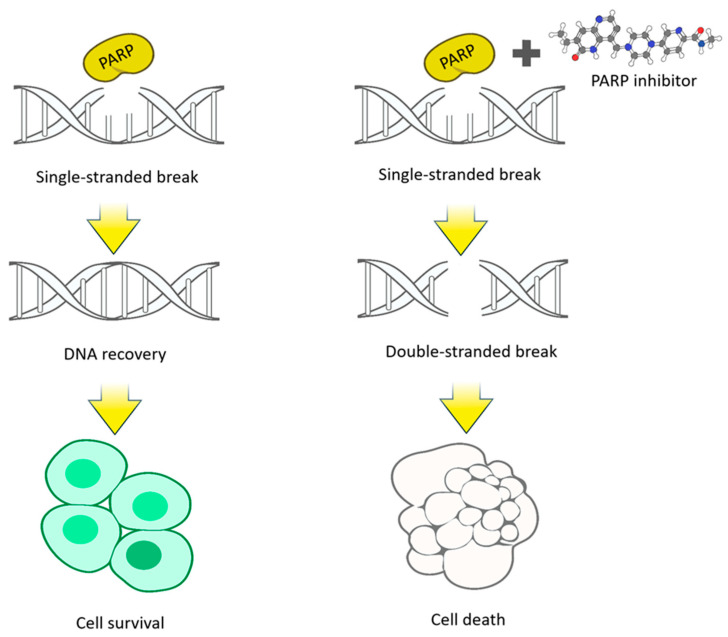
The mechanism of action of PARP inhibitors [6].

The critical role of PARP1 in DNA repair is reflected in its high expression levels in cancer cells [9]. Inhibition of PARP1 leads to the accumulation of DNA single-strand breaks (SSBs), which eventually convert into double-strand breaks (DSBs) (Figure 6). Because cancer cells often lack an efficient DSB repair mechanism such as homologous recombination (HR), PARP1 inhibition ultimately results in synthetic lethality. This mechanism underlies the importance of PARP1 as a target in the development of anticancer therapies [10,11].

In addition to simply blocking PAR synthesis, PARP inhibitors can also kill tumour cells through a “trapping” mechanism [12]. PARP inhibitors trap PARP on damaged DNA, leading to the accumulation of substantial amounts of PARP at sites of damage and thereby delaying its dissociation [13]. PARP–DNA trapping complexes exhibit greater cytotoxicity than unrepaired single-strand breaks resulting from PARP inactivation [14]. This suggests that PARP inhibitors function, at least in part, as agents that trap PARP enzymes on DNA.

**Figure 7:**
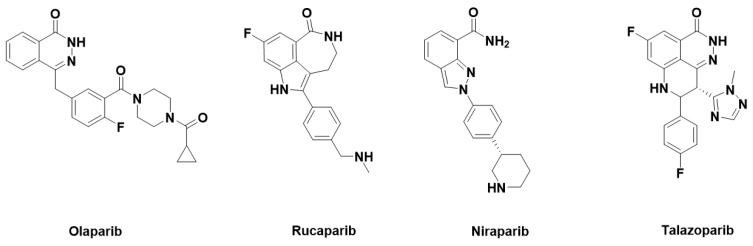
Non-selective PARP1 inhibitors approved for clinical use.

Olaparib (Figure 7), which acts on both PARP1 and PARP2, was approved in 2014 [15]. It was followed by the approval of several other PARP inhibitors, including rucaparib in 2016, niraparib in 2017, and talazoparib in 2018 (Figure 7). Like olaparib, these drugs exert dual activity against both PARP1 and PARP2 [16]. They are used across a wide range of indications.

Despite the clinical success of PARP1/2 inhibitors, these compounds have demonstrated significant toxic effects, most commonly hematological, including anemia, neutropenia, and thrombocytopenia [17,18]. These adverse effects are likely related to the low selectivity of these agents for PARP1 over PARP2, as they inhibit both enzymes [19]. This is supported by studies in knockout mice demonstrating an association between PARP2 and hematological toxicity [20]. Double knockout of PARP1 and PARP2 has also been shown to result in embryonic lethality [21]. Furthermore, studies by Murai et al. and Ronson et al. demonstrated that the synthetic lethality associated with BRCA mutations is mediated exclusively by PARP1, meaning that PARP2 trapping on DNA is not required for this effect [21]. In addition, loss of PARP2 has recently been shown to shorten red blood cell lifespan and impair erythroid progenitor differentiation, leading to chronic anemia [22].

In 2015, Nerviano Medical Sciences reported the PARP1-selective inhibitor NMS-P118 [23]. The company has now reported FDA approval of two Investigational New Drugs (INDs) for its PARP1-selective, BBB-penetrate inhibitor NMS-293. NMS-293 is a highly potent PARP-1 inhibitor with >200-fold selectivity over PARP-2 [24]. It is important to note that NMS-P118 is not a PARP trapping [25].

**Figure 8:**
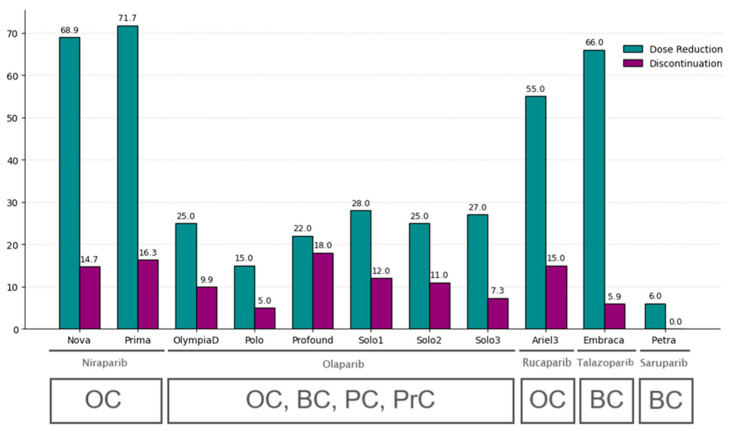
Dose reductions and treatment discontinuations of PARP inhibitor monotherapy in clinical trials. Under the names of the inhibitors are indicated the nosologies in which these compounds are registered (OC—ovarian cancer, BC—breast cancer, PC—prostate cancer, PrC—pancreatic cancer).

In 2021, AstraZeneca announced a PARP1 inhibitor with a selectivity index of 466 [26]. Saruparib marks a new stage in the development of PARP inhibitors, as selectivity has now become an important determinant of success. Preliminary results from the PETRA clinical trial of saruparib showed a significant reduction in the number of dose reductions and no treatment discontinuations at the 60 mg dose [27]. Compared with the reported rates of dose reductions and treatment discontinuations observed for non-selective inhibitors, saruparib appears more attractive for clinical use (Figure 8).

This new generation of inhibitors is expected to exhibit lower hematological toxicity and an improved therapeutic index [28]. This may benefit patients and also create new opportunities for combination strategies with other chemotherapeutic agents, where PARP1/2 inhibitors have previously shown limited utility because of overlapping toxicities. Reduced side effects may allow the use of next-generation inhibitors not only as monotherapy, but also in combination with radiotherapy. Ionizing radiation and conventional anticancer drugs induce DNA damage, and subsequent inhibition of PARP1 suppresses the repair of potentially lethal lesions, which may ultimately lead to cancer cell death [29]. AstraZeneca also introduced the PARP1 inhibitor palacaparib with a selectivity index of more than 19,000 [30]. Palacaparib is a BBB-penetrant inhibitor [31]. It is currently being evaluated in phase I/IIa trials both as monotherapy and in combination regimens.

**Figure 9:**
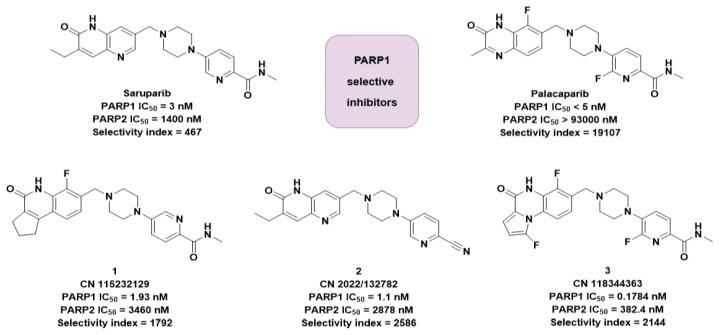
Structures of PARP1 selective inhibitors chosen for research.

In this study, we attempt to elucidate the mechanism of PARP1 selectivity using saruparib, palacaparib, and three highly selective molecules from our own companies (Figure 9). Palacaparib was selected as the most selective PARP1 inhibitor, demonstrating high activity and promising clinical potential. Saruparib, palacaparib, and three selected structures were included based on their similarity to saruparib and selectivity values greater than 1000. The Tanimoto similarity coefficient (TSC) was calculated for palacaparib and the three selected molecules using the saruparib reference protein. The TSC values were in the range of 0.5–0.7. Identifying patterns in the dynamics of selective and structurally similar ligands may allow us to propose a mechanism of selectivity. A similar method has already been used to study a selective CDK2 inhibitor in complexes with CDK1 and CDK2 [32]. The findings of that study contributed to the development of a highly selective CDK2 inhibitor [33]. A conceptually similar comparative molecular dynamics approach has also been applied to investigate clinically relevant differences among selective estrogen receptor modulators, linking ligand-specific interaction patterns in ER complexes to uterine cancer risk [34]. We therefore expect that the use of a similar approach may help clarify the mechanism underlying PARP1 selectivity.

## 2. Results

We compared the interaction patterns of the five ligands in both proteins to identify residues that may contribute to selective binding. For each residue pair, a *p*-value was calculated to determine whether the observed interaction frequencies in PARP1 and PARP2 differed significantly. We focused on residue pairs that (1) formed detectable interactions in all five complexes (PARP1 or PARP2), and (2) showed a nominally statistically significant difference in interaction frequency between PARP1 and PARP2 (*p* < 0.05). Such residue pairs were considered candidates for involvement in selective ligand binding to PARP1.

### 2.1. RMSD of Ligand-Protein Complexes

The quality of the prepared complexes for molecular dynamics simulations was assessed at each stage following temperature and pressure equilibration. The dynamic stability of the simulated complexes was monitored by analyzing the RMSD values of all backbone atoms and all ligand heavy atoms for each complex relative to the initial structure as a function of simulation time. In general, smaller RMSD fluctuations indicate greater complex stability.

**Figure 10:**
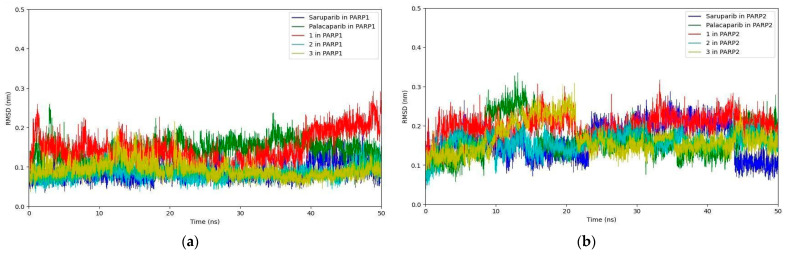
(**a**) RMSD of the ligand heavy atoms from the 50 ns MD simulations in PARP1. (**b**) RMSD of the ligand heavy atoms from the 50 ns MD simulations in PARP2.

As shown in Figure 10a, the RMSD profiles of ligand heavy atoms indicate stable behavior of all complexes over the course of the simulations. In PARP1 (Figure 10a), all systems reach a relatively stable regime after the initial equilibration phase (~5–10 ns), with RMSD values predominantly fluctuating within ~0.07–0.18 nm. Saruparib and compounds **2** and **3** exhibit the lowest fluctuations, maintaining stable plateau-like behavior throughout the trajectory. In contrast, compound **1** shows slightly higher variability and a gradual increase in RMSD toward the end of the simulation, reaching ~0.25–0.28 nm, suggesting reduced stability compared to the other systems.

In PARP2 (Figure 10b), the RMSD values are generally higher and exhibit more pronounced temporal fluctuations compared to PARP1. Most complexes fluctuate within ~0.12–0.25 nm; however, palacaparib and compound **3** display more prominent oscillatory behavior, characterized by sustained deviations around the mean rather than a well-defined plateau. These fluctuations are particularly evident in the 10–25 ns interval and persist throughout the trajectory, indicating increased conformational variability.

### 2.2. Statistical Analysis of Molecular Dynamics Trajectories

**Figure 11:**
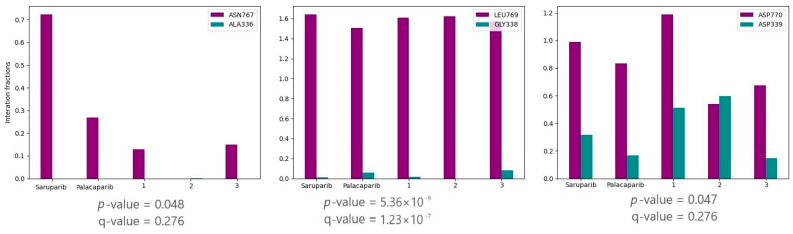
Interaction fractions of αF-helix residue pairs identified by nominal *p*-value screening. Leu769/Gly338 remained significant after Benjamini–Hochberg correction and represents the most statistically robust residue pair.

To identify the key residues involved in protein–ligand interactions, the obtained trajectories were analyzed using ProLIF. *p*-values were calculated for the resulting interaction fraction values. As shown in Figure 11, the amino acid pairs Asn767/Ala336, Leu769/Gly338, and Asp770/Asp339 exhibited *p*-values below 0.05. These results indicate that, in PARP1, selective inhibitors show higher interaction fraction values than in PARP2.

Additionally, q-values were calculated for all amino acid pairs using the Benjamini–Hochberg procedure to account for multiple comparisons. Only one amino acid pair, Leu769/Gly338, demonstrated a q-value below 0.05. This finding suggests that this specific pair represents the most statistically robust interaction and highlights Leu769/Gly338 as the key residue pair contributing to the observed selectivity.

**Figure 12:**
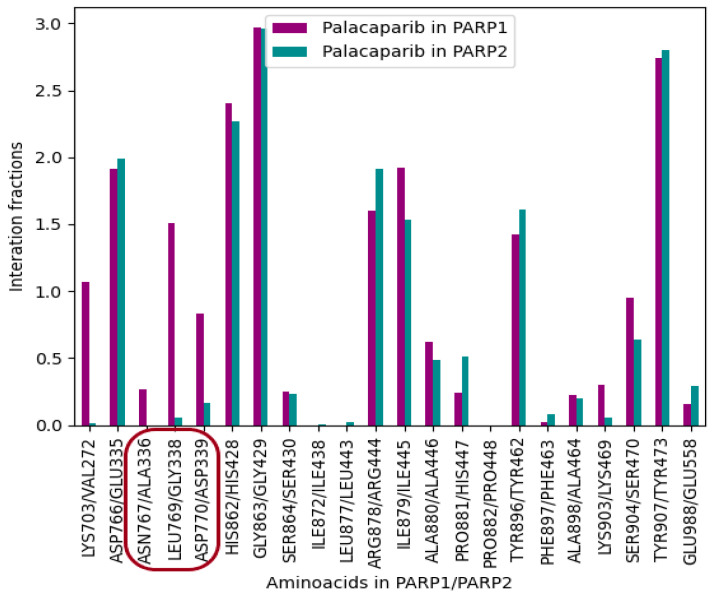
Fraction interactions of nominally significant αF-helix residue pairs in PARP 1/PARP 2. Adjacent amino acid residues showing nominal statistical significance are highlighted in red.

The key contacts characteristic of selective PARP1 inhibitors were investigated using palacaparib as the most selective inhibitor. Most corresponding amino acid residues in PARP1 and PARP2 retained similar interaction strengths (Figure 11). The exceptions were the amino acid pairs Asn767 in PARP1/Ala336 in PARP2, Leu769 in PARP1/Gly338 in PARP2, and Asp770 in PARP1/Asp339 in PARP2, which were identified by statistical analysis. For these residue pairs, the interaction fraction was lower in PARP2 than in PARP1 (Figure 11). The interaction fractions for palacaparib are presented in Figure 12.

As shown in Table 1, the interaction between palacaparib and Asn767 in PARP1 can be described as a van der Waals interaction, whereas palacaparib loses this interaction with the corresponding residue Ala336 in PARP2.

**Figure 13:**
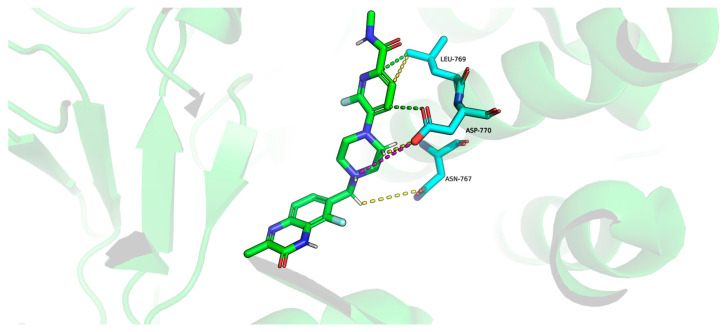
Interaction of amino acids Asn767, Leu769 and Asp770 (blue sticks) of PARP1 protein (green cartoon) with palacaparib (green). Van der Waals interactions are shown in yellow, hydrophobic interactions are shown in green, and cationic interactions are shown in purple.

**Figure 14:**
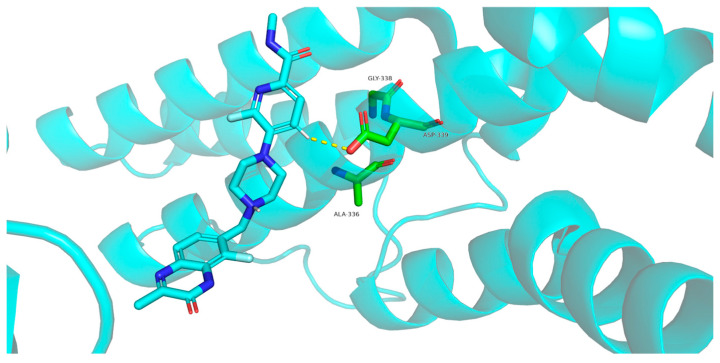
Interaction of amino acids Ala336, Gly338 and Asp339 (green sticks) of PARP2 protein (blue cartoon) with palacaparib (blue). Van der Waals interactions are shown in yellow.

A substantial part of the interaction between palacaparib and Leu769 in PARP1 is hydrophobic in nature and is also associated with van der Waals contacts. In PARP2, palacaparib loses the hydrophobic interaction with Gly338, while the van der Waals interaction is reduced tenfold. The interaction profile of palacaparib with the residue pair Asp770 in PARP1 and Asp339 in PARP2 changes markedly. In PARP1, van der Waals interactions, hydrophobic contacts, and a cation–π interaction are observed (Figure 13). In contrast, in PARP2, the hydrophobic contacts and the cation–π interaction are lost, and the van der Waals interaction is reduced by nearly fourfold (Figure 14).

## 3. Discussion

The issue of selectivity is fundamental to the development of PARP1 inhibitors, and researchers continue to investigate possible mechanisms and patterns that could explain selective binding. The first attempt to substantiate the mechanism of selectivity was reported in 2020. The authors performed a molecular dynamics study of the selective inhibitor NMS-P118 in complex with PARP1 and PARP2. Subsequent alanine scanning and MM/GBSA free energy calculations identified key residues responsible for selectivity.

**Figure 15:**
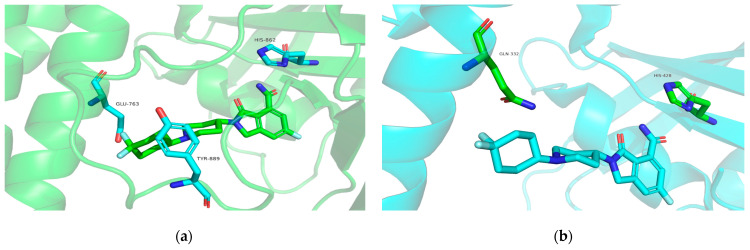
(**a**) Binding of NMS-P118 (green) and amino acid residues Glu763, His862 and Tyr889 (blue) in PARP1 (green cartoon). (**b**) Binding of NMS-P118 (blue) and amino acid residues Gln332 and His428 (green) in PARP1 (blue cartoon).

The results showed that most hotspot residues in PARP1 exhibited stronger binding free energies than the corresponding residues in PARP2. The 4,4-difluorocyclohexyl ring of NMS-P118 forms a favorable hydrophobic interaction with Tyr889 in PARP1 (Figure 15). In addition, residue His862 in PARP1 shows stronger binding free energy than His428 in PARP2 due to a shorter interaction distance and stronger hydrogen bonding. Moreover, the negatively charged residue Glu763 in PARP1 forms a stronger electrostatic interaction with positively charged NMS-P118 than the corresponding residue Gln332 in PARP2 [35].

**Figure 16:**
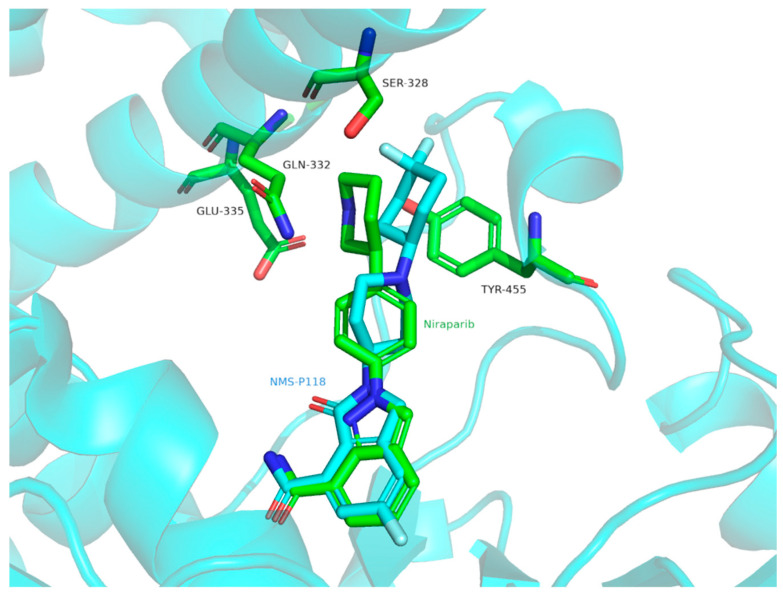
NMS-P118 (blue, PDB: 4ZZY) and Niraparib (green, PDB: 4R6E) overlay with amino acid residues Ser328, Gln332, Glu335 and Tyr455 (green) in PARP2 (blue cartoon).

A selectivity study using classical molecular dynamics and accelerated molecular dynamics was also performed with NMS-P118 and niraparib as examples of selective and non-selective PARP1 inhibitors, respectively [36]. The RMSD values of PARP1 Cα atoms showed only negligible fluctuations (<1 Å), indicating that NMS-P118 restricted the flexibility of the PARP1 protein structure. In contrast, the PARP2/NMS-P118 complex exhibited markedly larger fluctuations than the PARP1/NMS-P118 complex, indicating lower stability of PARP2 when bound to the selective PARP1 inhibitor NMS-P118. By comparison, the RMSD curves for niraparib in complexes with both PARP1 and PARP2 showed only minor fluctuations (<1 Å) during the last 400 ns of the simulation, suggesting that the non-selective inhibitor niraparib limited the structural flexibility of both proteins. Analysis of protein conformational distributions further showed that the PARP2/NMS-P118 complex sampled a broader conformational space than the PARP1/NMS-P118 complex. In contrast, PARP1/niraparib and PARP2/niraparib displayed a certain degree of similarity. These results indicate that binding of the selective inhibitor NMS-P118 affects the conformational flexibility of PARP1 and PARP2 differently, unlike the non-selective inhibitor niraparib. Importantly, residues Ser328, Gln322, Glu335, and Tyr455 formed significantly stronger polar interactions with niraparib than with NMS-P118 in PARP2 (Figure 16) [36]. Although the authors provided a detailed explanation of the possible basis of selectivity, these findings may not fully apply to the most selective PARP1 inhibitor reported to date, saruparib. NMS-P118 and saruparib differ substantially in structure and therefore are likely to achieve selectivity through different mechanisms.

Johannes et al., in their paper describing the discovery of palacaparib, proposed a possible mechanism of selectivity. Using saruparib as an example, they reported that the basic nitrogen atom in the central piperazine ring interacts with a highly organized water molecule in PARP1. They observed that selective PARP1 inhibitors contact this organized water molecule, which is also present in the crystal structures of PARP2, but is predicted to be considerably less energetically stable in PARP2. The authors therefore suggested that this ligand–water interaction plays an important role in selectivity [30].

**Figure 17:**
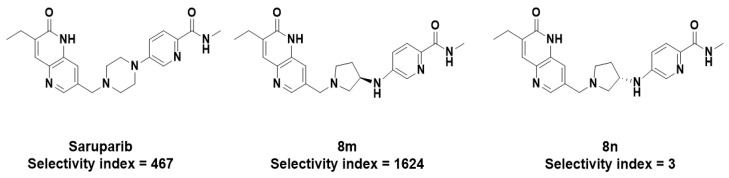
Structure of Saruparib and selective PARP1 inhibitors **8m** and **8n**.

This view is also supported by the work of other authors. Ren et al. developed a more selective PARP1 inhibitor, compound **8m** (selectivity index = 1261). Compound **8m** (Figure 17), like saruparib, interacted with water molecule 1262 and His862, and also formed an additional interaction with water molecule 1307 and Asp770, which may explain its substantial increase in activity and selectivity. In contrast, another compound, **8n** (Figure 17), lost the hydrogen bonds formed with water molecule 1262, which led to a marked decrease in activity against PARP1 [37].

However, this explanation of PARP1 selectivity based on the interaction of the piperazine ring with water does not provide a complete answer. Both saruparib and palacaparib contain a piperazine ring, yet palacaparib is substantially more selective than saruparib. This may indicate that preferential interaction with PARP1 is determined by several factors rather than by a single ligand–water contact.

Another proposed explanation for selectivity is based on subtle structural differences between PARP1 and PARP2. Comparative analysis of amino acid residues near the catalytic domains showed that the residues located in the αF-helix differ markedly between PARP1 and PARP2. In addition, the αF-helix is positioned slightly farther from the donor loop (D-loop) in PARP1 than in PARP2. This distinct orientation of the αF-helix, together with differences in amino acid composition, creates a potential selective site: a shallow pocket with a hydrophobic interior and hydrophilic edges. The larger and more hydrophobic environment of this site in PARP1 may provide an opportunity to develop selective PARP1 inhibitors with unique binding characteristics [38]. Bulky hydrophobic substituted phenyl groups are expected to be accommodated within this region.

**Figure 18:**
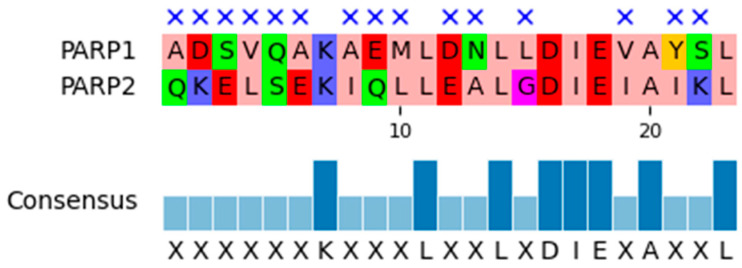
Alignment of the αF-helix for PARP1 and PARP2. The cross marks the mismatched amino acid residues. The consensus sequence represents the most common amino acid variant at a given position for the two proteins.

**Figure 19:**
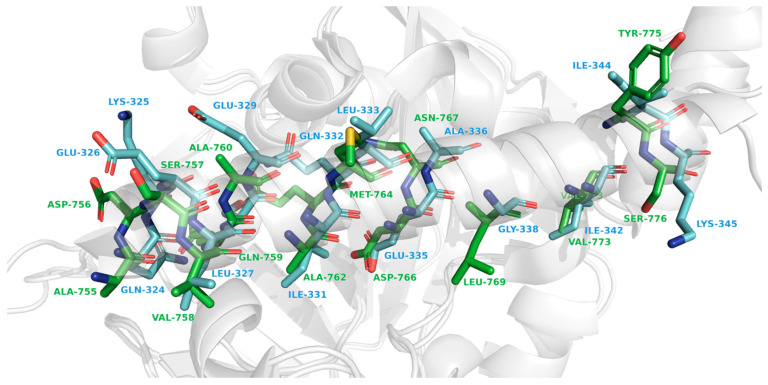
Structure of αF-helix. PARP1 amino acid residues are shown in green, PARP2 amino acid residues are shown in blue.

Our findings closely align with this proposed model. The strongest statistically supported difference was observed for Leu769/Gly338, a residue pair located in the αF-helix near the catalytic domain. Importantly, the neighboring residue pairs Asn767/Ala336 and Asp770/Asp339 also showed nominal interaction differences and consistent loss or weakening of contacts in PARP2 (Figure 18). Differences in amino acid sequence are accompanied by differences in the spatial organization of the αF-helix. The corresponding residues in PARP2 are bulkier and occupy more space (Figure 19), and the close proximity of the αF-helix to the ligand-binding site suggests that this structural element plays an important role in the selectivity of PARP inhibitors.

As shown by our molecular dynamics data for selective inhibitors, hydrophobic contacts and van der Waals interactions play a significant role in selectivity. Hydrophobic contacts are among the most common interactions in protein–ligand complexes [39] and contribute to ligand stabilization at the target, thereby enhancing binding affinity and drug potency [40]. Van der Waals interactions are weaker, and their strength decreases rapidly as the ligand moves away from the protein. Owing to differences in amino acid composition, the α-helix in PARP1 and PARP2 is likely positioned at different distances from the binding site, as reflected by the reduced strength and number of van der Waals interactions in PARP2.

First, it is important to identify which parts of palacaparib interact with the detected amino acids. Asn767 forms a van der Waals interaction with the hydrogen attached to the carbon atom located between the core nicotinamide mimic and the piperazine core. Asp770 forms a cation–π interaction with the charged nitrogen of the piperazine ring and a hydrophobic interaction with the pyridine core. Leu769 shows both van der Waals and hydrophobic interactions with the pyridine core. In PARP2, only Asp339, the residue corresponding to Asp770 in PARP1, retains a van der Waals interaction with the pyridine core. These observations suggest that, in most cases, the pyridine core interacts with the α-helix. It can therefore be assumed that, to increase activity and selectivity toward PARP1, it may be advantageous to place a bulky group in this part of the molecule instead of the pyridine core.

**Figure 20:**
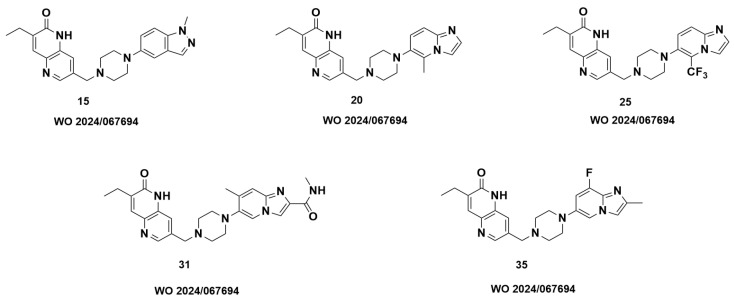
Structures from the patent WO 2024/067694.

**Figure 21:**
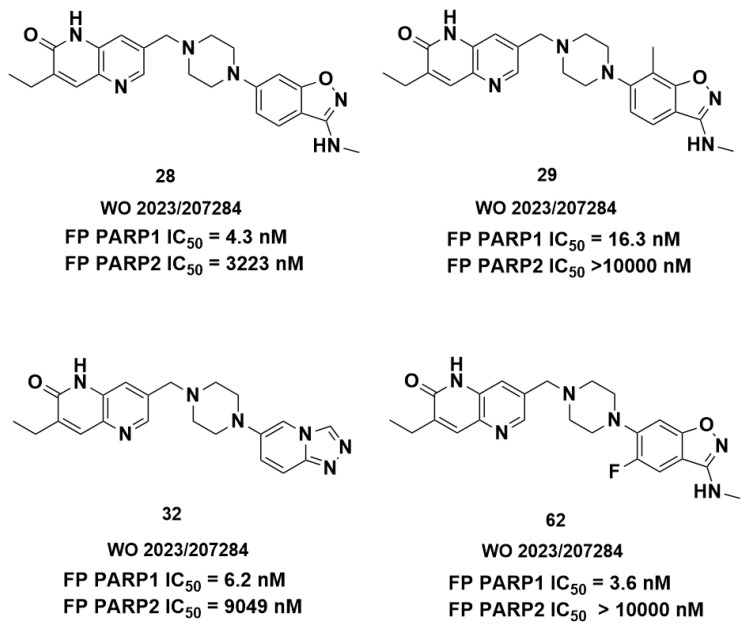
Structures from the patent WO 2023/207284.

To test this hypothesis, we examined patents on selective PARP1 inhibitors in search of saruparib-like structures containing a bicyclic ring in place of the pyridine ring. Very few such structures were identified; however, we found two patents in which the pyridine is replaced by a 6,5-bicyclic system. The representative structures selected as examples exhibited both activity and selectivity toward PARP1. For example, structures from patent WO2024/067694 (Figure 20) showed IC_50_ values below 10 nM for PARP1, whereas the IC_50_ values for PARP2 were above 10,000 nM. Similar features can be observed in WO 2023/207284. The structures of the inhibitors and their activities against PARP1 and PARP2 are shown in Figure 21. These data indicate that compounds containing a 6,5-bicyclic system exhibit a high degree of selectivity. For compounds **32** and **62** the selectivity index is 1460 and 2778, respectively.

**Figure 22:**
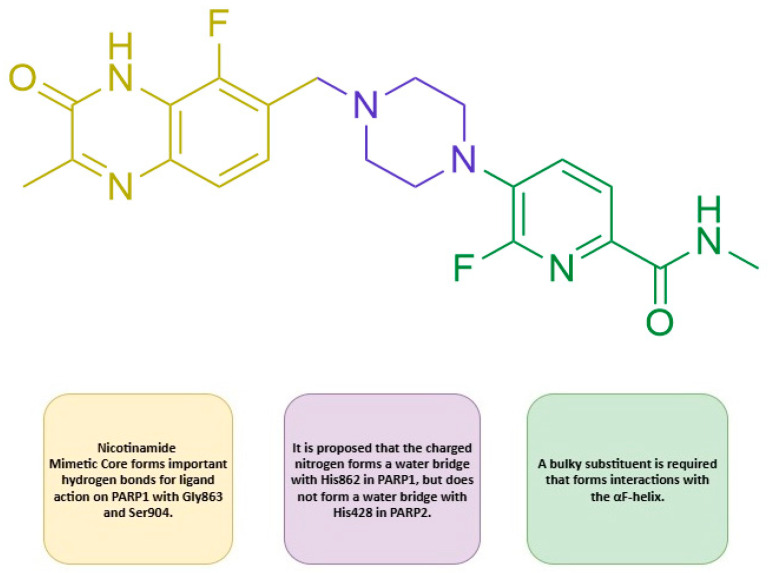
Template for the creation of an active and selective PARP1 inhibitor.

Based on the available literature data and the results of our molecular dynamics simulations, we can propose a model design for a selective and highly active PARP inhibitor (Figure 22). The nicotinamide core forms the basis of inhibitor activity, as this fragment is required for the formation of hydrogen bonds with Gly863 and Ser904. The next important fragment is the piperazine moiety. Published data suggest that the charged nitrogen atom plays a major role in inhibitor selectivity by forming a water bridge with His862. This indicates that, to preserve or enhance selectivity, it is reasonable to retain a heterocycle containing a charged nitrogen in this part of the molecule. Another important part of the molecule is the pyridine core.

## 4. Materials and Methods

Multiple sequence alignment of human PARP1 and PARP was performed by Clustal Omega (version 1.2.4) [41] and visualized using the pyMSAviz tool (version 0.5.0) [42], allowing comparative analysis of conserved domains. Structural models of protein–ligand complexes were visualized and superimposed using PyMOL (version 2.5) [43].

Crystal structures of human PARP1 (PDB ID: 7ONT) and PARP2 (PDB ID: 4TVJ) were obtained from the RCSB Protein Data Bank [44]. The 3D structures of saruparib were retrieved from PubChem. Palacaparib and the patent structures were generated from the saruparib structure by editing the molecule using Builder in Pymol (version 2.5).

As no experimental structure of PARP2 bound to saruparib was available, molecular docking was performed using smina (version 2020.12.10) [45] with default parameters. The resulting docking poses were evaluated based on scoring and binding geometry, and the highest-ranked, structurally plausible complex was selected for molecular dynamics simulations. As with the patent structures in PARP1, palacaparib and other investigational compounds were generated in PARP2 from saruparib using the Pymol Builder.

Molecular dynamics simulations were conducted using the GROMACS software (version 2024.4) suite [46]. Protein topologies were generated using the AMBER force field, and ligand topologies were constructed using AMBER [47]. Each protein–ligand complex was placed in a cubic simulation box filled with TIP3P water molecules, and appropriate counterions were added to neutralize the system. Energy minimization was followed by two equilibration phases: first under constant volume and temperature (NVT ensemble) for 100 ps, and then under constant pressure and temperature (NPT ensemble) for another 100 ps.

Production molecular dynamics simulations were performed for 50 nanoseconds per complex with a 2-femtosecond time step. To ensure statistical reliability and reproducibility, three independent simulations were conducted for each protein–ligand pair. All MD trajectories were post-processed and analyzed using the ProLIF Python package (version 2.0.3) [48] to quantify non-covalent ligand–protein interactions, including hydrogen bonding, π-stacking, and hydrophobic contacts. To directly compare PARP1 and PARP2, the structures of the two proteins were structurally aligned and the corresponding residue pairs were identified based on the spatial correspondence in the binding site. For each aligned pair, the total interaction contribution was calculated as the sum of all observed non-covalent interactions during each simulation. Visualization of interaction patterns and dynamic changes over time was performed using Matplotlib (version 3.10.5) [49]. Statistical processing of the MD results is carried out using the scipy.stats package (version 1.16.1) [50]. The statistical analysis was performed using an independent samples *t*-test (Student’s *t*-test). To account for multiple comparisons across aligned residue pairs, *p*-values were further adjusted using the Benjamini–Hochberg procedure, and q-values below 0.05 were considered statistically significant after false discovery rate correction. Nominal *p*-values below 0.05 were used only as an exploratory screening criterion.

Molecular similarity analysis was performed to select compounds with moderate structural similarity to the reference molecule, while avoiding both structurally unrelated compounds and near-duplicate analogues. SMILES representations were converted into molecular fingerprints using the Morgan algorithm as implemented in RDKit [51]. A fingerprint radius of 1 and a bit vector size of 2048 were used to emphasize local atomic environments and functional group similarity.

Pairwise molecular similarity was quantified using the Tanimoto coefficient relative to the Saruparib structure. Compounds with Tanimoto coefficient values between 0.5 and 0.7 were selected for further analysis. The lower threshold of 0.5 was used to ensure that the selected molecules retained sufficient structural similarity to Saruparib and therefore remained within the relevant chemical space for comparative molecular dynamics analysis. The upper threshold of 0.7 was applied to avoid selecting highly similar near-duplicate analogues, which could reduce structural diversity and limit the generalizability of the observed interaction patterns. Tanimoto coefficient values are presented in Table 2.

Schematic visualization is made using BioArt Collection, visualization in the form of diagrams is made using draw.io (version 28.0.6) [52] and Matplotlib.

## 5. Conclusions

A new generation of selective PARP1 inhibitors opens up new opportunities for the treatment of various oncological diseases. A reduction in the frequency and severity of adverse effects, particularly hematotoxicity, together with the absence of treatment discontinuation observed in clinical trials of saruparib, suggests that selectivity toward PARP1 is one of the most important criteria in the development of new inhibitors. Increased selectivity may enable effective targeting of tumor cells while minimizing inhibition of related enzymes associated with undesirable toxic effects.

The selectivity of PARP1 inhibitors is determined by several structural and dynamic factors that influence the stability of ligand–protein complexes. In our study, we sought to investigate the molecular basis of this selectivity using molecular dynamics simulations of selective inhibitors in complexes with PARP1 and PARP2. Comparative analysis of the interaction profiles identified Leu769/Gly338 as the most statistically robust residue pair associated with reduced interaction frequency in PARP2 after correction for multiple comparisons. In addition, the neighboring αF-helix residue pairs Asn767/Ala336 and Asp770/Asp339 showed nominally reduced interaction frequencies and consistent contact weakening in the palacaparib complex. Together, these observations support a model in which PARP1 selectivity is not driven by a single isolated residue, but by a Leu769-centered interaction pattern within the αF-helix region.

Such differences in the αF-helix may play an important role in shaping the binding environment of selective inhibitors. Our results suggest that the introduction of a bulky structural group into the pyridine fragment of the inhibitor molecule may enhance interactions with PARP1 while simultaneously reducing favorable contacts with PARP2. This structural modification could exploit the unique spatial arrangement of residues in the PARP1 αF-helix, thereby contributing to improved selectivity.

Furthermore, molecular dynamics simulations indicate that differences in local flexibility and interaction stability within this region may additionally contribute to selective ligand binding. The proposed mechanism is consistent with available experimental and biological data reported in patents describing highly selective PARP1 inhibitors with similar structural features. Taken together, our findings provide additional insight into the molecular determinants of PARP1 selectivity and may serve as a useful basis for the rational design of next-generation PARP inhibitors with improved therapeutic efficacy and reduced toxicity.

## Figures and Tables

**Table 1:** Fractions of interaction between palacaparib and PARP1/PARP2 for selected αF-helix residue pairs.

Amino Acid Pair	Type of Interaction	Interaction Fraction in PARP1	Interaction Fraction in PARP2
Asn767/Ala336	Van der Waals contact	0.269	0
Leu769/Gly338	Hydrophobic contact	0.994	0
Leu769/Gly338	Van der Waals contact	0.513	0.058
Asp770/Asp339	Hydrophobic contact	0.044	0
Asp770/Asp339	Cationic contact	0.136	0

**Table 2:** Tanimoto coefficient values for selected structures compared with saruparib.

Compound	Tanimoto Coefficient
Palacaparib	0.605
**1**	0.667
**2**	0.692
**3**	0.568

## Data Availability

The original contributions presented in this study are included in the article. Further inquiries can be directed to the corresponding author.
